# Real-World Outcomes of Immediate Femoral Sheath Removal After Emergency Embolization in the Age of Ultrasound-Guided Device-Assisted Vascular Closure

**DOI:** 10.3390/diagnostics16010040

**Published:** 2025-12-22

**Authors:** Terrence Hui, Akshay Kohli, Ross Copping, Hannah Ireland, Shady Osman, Bryan Barry, Jules Catt, Glen Schlaphoff

**Affiliations:** 1 Department of Diagnostic Radiology, Tan Tock Seng Hospital, Singapore 308433, Singapore; 2 Department of Interventional Radiology, Liverpool Hospital, Liverpool, NSW 2170, Australia; akshayk.livrad@gmail.com (A.K.); ross.copping@health.nsw.gov.au (R.C.); hannah.ireland1@health.nsw.gov.au (H.I.); shady.osman@health.nsw.gov.au (S.O.); bryan.barry@health.nsw.gov.au (B.B.); jules.catt@health.nsw.gov.au (J.C.); glen.schlaphoff@health.nsw.gov.au (G.S.); 3 Discipline of Medicine, UNSW Medicine and Health, Sydney, NSW 2052, Australia

**Keywords:** access site complications, emergency embolization, endovascular, trauma, vascular closure device

## Abstract

**Background/Objectives**: Emergency arterial embolization is a life-saving procedure typically performed via femoral access. This study evaluated the safety and efficacy of immediate femoral sheath removal following emergency embolization. **Methods**: A retrospective cohort study was conducted at a Level 1 trauma center (January 2022–May 2025). Adult patients undergoing emergency embolization with immediate sheath removal were included. Endpoints were reintervention (repeat embolization within 7 days) and access site complications. Multivariate logistic regression identified independent predictors of outcomes. **Results**: A total of 322 emergency embolization procedures in 299 patients were included. The most common indication was gastrointestinal hemorrhage (45.7%). Vascular closure devices (VCDs) were used in 92.5% of cases. The re-intervention rate was 4.0% (13/322). The overall access site complication rate was 6.2% (20/322), with a major complication rate of 0.9% (3/322). On univariate analysis, pre-procedural platelet level ≤ 80 × 10^9^/L (*p* = 0.034) and INR > 1.5 (*p* = 0.034) were significantly associated with an increased risk of complications. On multivariate analysis, pre-procedural platelets ≤ 80 × 10^9^/L was the strongest independent predictor of access site complications (OR 7.28, 95% CI 1.51–35.12; *p* = 0.013). Choice of vascular closure device was an independent predictor for both reintervention and complications (*p* < 0.05), likely reflecting bias. **Conclusions**: Immediate femoral sheath removal following emergency embolization is safe for most patients. However, thrombocytopenia is a significant risk factor for access site complications. A risk-stratified approach with consideration for delayed sheath removal is warranted for patients with platelet counts ≤ 80 × 10^9^/L.

## 1. Introduction

Emergency arterial embolization is a minimally invasive, life-saving procedure performed by Interventional Radiologists for controlling acute haemorrhage [1,2,3,4,5,6,7,8,9,10]. This technique is an important alternative to emergency surgery for various clinical scenarios such as acute gastrointestinal bleeding and solid organ injury, and the common femoral artery (CFA) is often used as the site of primary vascular access [9,10,11,12,13,14].

While immediate sheath removal is standard practice in elective cases, the practice in emergency scenarios varies. Interventionalists may opt to leave vascular sheaths in situ after the procedure temporarily, closing the access after a few days of observation. The main reasons for this conservative practice are patient instability, coagulopathy and anticipation for a re-look angiogram and/or re-embolization [11]. However, this cautious approach is not without its own risks and costs. Indwelling arterial sheaths are foreign body objects with the potential to cause access site infection, arterial thrombosis and distal limb ischemia [15]. Prolonged indwelling arterial sheaths also extends patient immobilization, requiring additional nursing care and continuous saline flushing to maintain patency.

The use of vascular closure devices (VCDs) has significantly changed access site management over the past decade, allowing the interventionalist to achieve rapid and reliable haemostasis, improving patient comfort and reducing time-to-ambulation [16,17,18,19,20]. Özyurtlu et al. found that there is no difference in complication rates between immediate and delayed sheath removal after percutaneous coronary intervention, but better patient comfort with early removal [21]. Yet there is a critical knowledge gap regarding sheath management strategy for patients undergoing emergency embolization in the interventional radiology suite. To our knowledge, no study to date has addressed sheath management in this specific group of patients.

At our institution, immediate sheath removal, often with VCDs, is standard of care following emergency embolization, offering a unique opportunity to evaluate real-world outcomes of such a strategy. This study aims to evaluate patient outcomes associated with immediate femoral sheath removal following emergency embolization procedures by assessing reintervention and access site complication rates.

## 2. Materials and Methods

This is a retrospective, cohort study conducted at a large, academic Level 1 trauma center. Human Ethics Application for this retrospective study was approved (2025/ETH01056) on 3 July 2025. Due to the retrospective nature of this study, the requirement for informed consent was waived by the Institutional Review Board under the ‘Low or negligible risk review pathway’. All patient data were de-identified and anonymized prior to analysis to ensure confidentiality. All adult patients (18 years and above) who underwent emergency embolization procedures in the Interventional Radiology suite via femoral access between 1 January 2022 and 31 May 2025 were included. Exclusion criteria were elective nature of the procedure, primary vascular access via a non-femoral route such as radial artery, loss to follow-up or death unrelated to access site complications within 7 days (168 h), and incidents where vascular sheaths were left in situ.

For all cases, CFA access was obtained with Seldinger technique under real-time ultrasound (US) guidance in accordance with CIRSE guidelines [22]. Following completion of the procedure, hemostasis was achieved, most often with a VCD. The choice of VCD used was left to the discretion of the performing Interventional Radiologist. Deployment of all VCDs was performed under strict aseptic conditions and real-time ultrasound guidance to ensure device position and adequate device–vessel wall apposition [23,24,25].

Following the procedure, all patients were transferred to a ward or intensive care unit (ICU) as determined by the clinical team. The standard institutional protocol mandates complete rest in bed for 4 h following VCD closures and 6 h following manual compression (MC). The access sites and ipsilateral limb neurovascular status were assessed and findings documented in the electronic medical record by IR medical staff and ward nurses. The access sites were evaluated for bleeding, ecchymosis, hematoma formation, signs of infection, and pseudoaneurysm formation. The distal neurovascular status of the ipsilateral limb was also assessed for distal pulses, temperature, color and sensation. The results of any imaging which included the access site performed after embolization are reviewed.

Endpoints of this study were reintervention rates and access site complication rates. A re-intervention procedure was defined as clinically significant re-bleeding requiring a repeat emergency embolization procedure performed for the same indication as the index procedure within 7 days (168 h). Minor access site complications were defined as access site bleeding treated conservatively, hematoma formation < 5 cm and ecchymosis (bruising). Major complications are defined as hematoma formation ≥ 5 cm, pseudoaneurysm formation, retroperitoneal hematoma formation, access site infection and distal limb ischemia.

All statistical analyses were performed with Statistical Package for Social Sciences (IBM SPSS Statistics for Mac, Version 30.0. Armonk, NY, USA). The Kolmogorov–Smirnov test was used to determine if the continuous variables were distributed normally. Continuous variables are presented as median (interquartile range) and categorical variables as numbers (percentages). Non-normally distributed continuous variables were compared with the Mann–Whitney U test. Categorical variables were analyzed using the Chi-square or Fisher’s exact test as appropriate. To identify independent predictors of reintervention and access site complications, multivariate binary logistic regression was performed; variables with a *p*-value of <0.25 on univariate analysis were included in multivariate analysis. Adjusted odds ratios (OR) with 95% confidence intervals (CI) were reported. A *p*-value of <0.05 is considered to be statistically significant.

## 3. Results

A total of 322 procedures performed on 299 patients were included in our study. The median age of the patients was 66.0 years (interquartile range [IQR] 26.0); the majority were male (68.9%; *n* = 209) and they had a median body mass index (BMI) of 26.6 kg/m^2^ (IQR 8.5). Obesity (BMI ≥ 30 kg/m^2^) was present in 29.1% (*n* = 87) of patients. Current or former smoking was reported in 20.7% (*n* = 62). Diabetes mellitus (DM) was documented in 24.7% (*n* = 74), hypertension (HTN) in 46.8% (*n* = 140), coronary artery disease (CAD) in 16.7% (*n* = 50), and peripheral vascular disease (PVD) in 4.3% (*n* = 13). Antithrombotic drug use was common, with 63.5% (*n* = 190) receiving none, 17.4% (*n* = 52) on anticoagulation, 11.4% (*n* = 34) on single antiplatelet therapy, 4.0% (*n* = 12) on both single antiplatelet and anticoagulation, and 3.3% (*n* = 10) on dual antiplatelet therapy, with an additional 0.3% (*n* = 1) on dual antiplatelet and anticoagulation.

The most frequent indications for intervention were lower GI hemorrhage (*n* = 85, 26.4%) and upper GI hemorrhage (*n* = 62, 19.3%). Solid organ and visceral bleeding included splenic (*n* = 39, 12.1%), renal (*n* = 30, 9.3%), hepatic (*n* = 28, 8.7%), and pelvic hemorrhage (*n* = 25, 7.8%), as well as hemoptysis (*n* = 28, 8.7%). Less common indications comprised bleeding from the lumbar artery (*n* = 9, 2.8%), inferior epigastric artery (*n* = 7, 2.2%), or intercostal artery (*n* = 4, 1.2%), and extremity hemorrhage (*n* = 3, 0.9%). Polytrauma accounted for two cases (0.6%).

Vascular access was established via the right common femoral artery (CFA) in 298 cases (92.5%) and the left CFA in 24 cases (7.5%). The 5-French sheath was the predominant size used (*n* = 293, 91.0%), followed by 6-French (*n* = 14, 4.3%), 4-French (*n* = 10, 3.1%), and 7-French (*n* = 5, 1.6%).

Embolisation was performed in 296 cases (91.9%) and not performed in 26 cases (8.1%). Regarding embolic selection, coils were used as monotherapy in 148 cases (46.0%). Combination therapies involving coils included coils with Gelfoam (*n* = 19, 5.9%), coils with particles (*n* = 16, 5.0%), coils with glue (*n* = 12, 3.7%), and coils with an Amplatzer plug (*n* = 6, 1.9%). Other monotherapies utilized were particles only (*n* = 42, 13.0%), glue only (*n* = 36, 11.2%), and plugs only (*n* = 3, 0.9%). Rare combinations included glue with Gelfoam (*n* = 1, 0.3%), glue with particles (*n* = 1, 0.3%), and glue with a plug (*n* = 1, 0.3%). Two cases (0.6%) required more than three embolic agents.

Access site closure was achieved primarily using AngioSeal (Terumo Interventional Systems, Somerset, NJ, USA; *n* = 232, 72.0%) and Mynx Control (Cordis US Corporation, Miami Lakes, FL, USA; *n* = 60, 18.6%). Other devices included Perclose Proglide (Abbott Vascular, Santa Clara, CA, USA; *n* = 5, 1.6%) and Starclose SE (Abbott Vascular, Santa Clara, CA, USA; *n* = 1, 0.3%). Manual compression (MC) was utilized in 24 cases (7.5%). Post-procedure, 45.0% of patients (145/322) were admitted to high-dependency/intensive care units for post-procedure care.

### 3.1. Reintervention

**Figure 1:**
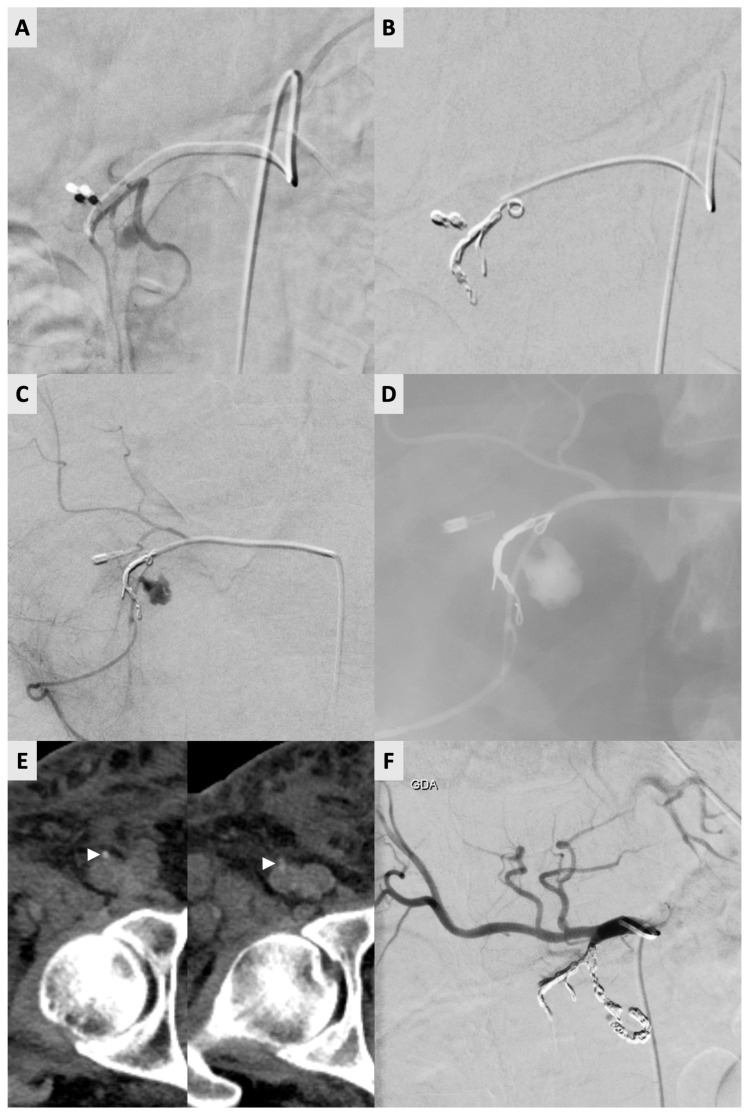
A 60-year-old male undergoing endoscopic ultrasound-guided fine needle aspiration (EUS-FNA) for a pancreatic mass, complicated by duodenal hemorrhage, requiring angiographic intervention. (**A**) Initial angiogram revealing a pseudoaneurysm of the posterior superior pancreaticoduodenal artery (PSPDA). (**B**) Successful coil embolization of the gastroduodenal artery (GDA) and PSPDA was performed with favorable angiographic outcome. Hemostasis was achieved with 6F AngioSeal. (**C**) Recurrent symptoms 4 days after index procedure prompted a repeat angiogram confirming recanalization of the pseudoaneurysm. (**D**) Subsequent embolization of the pseudoaneurysm using glue was performed. Hemostasis again achieved with 6F AngioSeal. (**E**) Symptoms recurred 3 days later, and a follow-up CTconfirmed re-bleeding. Unenhanced CT of the right common femoral artery (CFA) showing two closely positioned AngioSeal devices (white arrowheads) seen as small high-density structures within or adjacent to the previous puncture. (**F**) Re-repeat definitive embolization of GDA branches with coils to address persistent bleeding was performed and patient’s bleeding symptoms resolved. Hemostasis was again achieved with 6F AngioSeal, the 3rd deployment in the same vessel in 8 days.

Reintervention was required in 13 of 322 (4.0%) procedures (Table 1). One of these cases required two reinterventions. For the 13 re-intervention procedures, the ipsilateral right CFA was used again in 11 out of 13 occasions (83.3%). The contralateral left CFA was used on two occasions (2/12) due to small hematoma formations (<5 cm) at the previous vessel puncture site. For the re-intervention procedures with arterial access on the ipsilateral CFA, AngioSeal (*n* = 7), Mynx Control (*n* = 4) and manual compression (*n* = 2) were used for closure during the index procedure. Notably, the abovementioned patient required three right CFA accesses (and three Angio-Seal deployments) within an 8-day period (Figure 1).

Univariate analysis identified no statistically significant predictors of reintervention: age (*p* = 0.353), gender (*p* = 0.277), BMI (*p* = 0.655), obesity (*p* = 0.971), significant smoking history (*p* = 0.154), history of DM (*p* = 0.383), history of HTN (*p* = 0.778), history of CAD (*p* = 0.342), history of PVD (*p* = 0.296), pre-procedural antithrombotic therapy (*p* = 0.061), pre-procedural hemoglobin ≤ 100 g/L (*p* = 0.286), platelet count ≤ 80 × 10^9^/L (*p* = 0.647), INR > 1.5 (*p* = 0.105), procedural indication (*p* = 0.915), performance of embolization (*p* = 0.323) or choice of VCD (*p* = 0.238).

Multivariate analysis was performed with multivariate binary logistic regression to identify independent predictors of reintervention, controlling for smoking history, closure device, use of blood thinners and INR. The choice of VCD used was a significant independent predictor of reintervention (*p* < 0.05). Specifically, the use of MC (OR 7.18, 95% CI 1.18–43.82, *p* = 0.033) and Mynx (OR 4.20, 95% CI 1.12–15.78, *p* = 0.034) was associated with higher odds of reintervention compared to AngioSeal. Elevated INR > 1.5 (OR 3.55, 95% CI: 0.84–14.92, *p* = 0.084) and antithrombotic use (OR 2.74, 95% CI: 0.79–9.49, *p* = 0.112) were not found to be significant but displayed a strong trend towards re-intervention. Smoking history was not found to be statistically significant (*p* = 0.335).

### 3.2. Complications

The overall access-site complication rate was 6.2% (20/322 procedures). Minor complications occurred in 17 patients (5.3%) and comprised prolonged oozing (*n* = 7), ecchymosis (*n* = 6), and small hematoma (<5 cm; *n* = 4). Major complications occurred in three patients (0.9%): pseudoaneurysm requiring ultrasound-guided thrombin injection (*n* = 2) and retroperitoneal hemorrhage requiring transfusion (*n* = 1).

On univariate analysis, pre-procedural platelet count ≤ 80 × 10^9^/L (*p* = 0.034) and INR >1.5 (*p* = 0.034) were significantly associated with complications (Table 2). Age (*p* = 0.246), gender (*p* = 0.489), BMI (*p* = 0.555), obesity (*p* = 0.481), smoking history (*p* = 0.584), history of DM (*p* = 0.606), history of HTN (*p* = 0.796), history of CAD (*p* = 0.092), history of PVD (*p* = 0.573), sheath size (*p* = 0.789), type of VCD (*p* = 0.106), pre-procedural antithrombotic therapy (*p* = 0.442), pre-procedural hemoglobin ≤100 g/L (*p* = 0.779), and pre-procedural estimated glomerular filtration rate < 60 mL/min/1.73 m^2^ (*p* = 0.260) did not reach statistical significance.

Multivariate analysis was performed with multivariate binary logistic regression to identify independent predictors of access site complications, controlling for age, coronary artery disease, closure device, platelet count and INR. Pre-procedural thrombocytopenia (platelets ≤ 80 × 10^9^/L) was identified as the strongest independent predictor of complications (OR 7.28, 95% CI 1.51–35.12; *p* = 0.013). The choice of VCD was also a significant independent predictor, with Mynx associated with higher complication rates compared to AngioSeal (OR 3.43, 95% CI 1.23–9.57; *p* = 0.018). While pre-procedural INR > 1.5 showed a trend towards increased risk (OR 2.78), it did not reach statistical significance in this model (*p* = 0.089). Age (*p* = 0.490) and history of CAD (*p* = 0.202) were not significant predictors.

## 4. Discussion

This study demonstrates that immediate femoral sheath removal after emergency embolization, facilitated by ultrasound-guided access and predominantly vascular closure device (VCD) deployment, is associated with low rates of reintervention (4.0%) and access-site complications (overall 6.2%, major 0.9%). The reported rate of complication was in line with adverse events reported in the literature. Das et al. reported CFA access complication rate ranges between 3.1% and 11.4% in a meta-analysis of 21 studies, featuring 3662 patients undergoing non-coronary interventions [26]. In a similar subset of patients, Adnan et al. report an overall complication rate of 9.9% in trauma patients undergoing angioembolization through the CFA [27]. Additionally, we found that the decision to embolize or not did not predict the need for reintervention (*p* = 0.323). This suggests that even in cases where angiography did not show a ‘bleeder’ and no embolization was performed, the sheath can be removed at the end of the procedure without an increased need for re-intervention. With ultrasound-guided puncture and closure with VCDs, the professed risks of immediate vascular access closure may be mitigated, with a vast majority of patients not requiring re-intervention.

On multivariate analysis, neither pre-procedural antithrombotic drug use (OR 2.74, 95% CI: 0.79–9.49, *p* = 0.112) nor INR > 1.5 (OR 3.55, 95% CI: 0.84–14.92, *p* = 0.084) emerged as independent predictors of reintervention within 7 days. These variables had shown borderline association on univariate testing (*p* = 0.061 and *p* = 0.105, respectively). This is most likely to reflect the low absolute number of reinterventions (*n* = 13), and a clinically relevant increase in risk of requiring reintervention cannot be definitively excluded.

On the other hand, we found that the choice of VCD used was a significant independent predictor of reintervention on multivariate analysis (*p* < 0.05). The use of MC (OR 7.18, 95% CI 1.18–43.82, *p* = 0.033) and Mynx (OR 4.20, 95% CI 1.12–15.78, *p* = 0.034) was associated with higher odds of reintervention compared to AngioSeal. This finding likely reflects a confounding by indication bias; Interventional Radiologists may opt to use the Mynx Control or manual compression for closure if they anticipate a higher likelihood of re-intervention. Mynx Control achieves hemostasis using an extravascular sealant with no intravascular component, and this design is marketed as safe for immediate re-puncture. AngioSeal, on the other hand, uses a bioabsorbable footplate and its manufacturer recommends re-puncture of an AngioSeal site 60–90 days later to allow for full absorption of the intravascular anchor.

We found that the choice of VCD was a significant independent predictor of access site complications, with Mynx associated with higher complication rates (13.3% versus 5.2%) compared to AngioSeal (OR 3.43, 95% CI 1.23–9.57; *p* = 0.018). However, due to the abovementioned confounding by indication bias, a definitive conclusion cannot be made. Previous studies have shown AngioSeal and Mynx Control devices to have comparable complication rates [28,29].

Our experience showed that re-access of a CFA previously closed with AngioSeal is feasible and safe when performed with US guidance to avoid ‘hitting’ the pre-existing indwelling anchor. This practice is in line with the device IFU which recommends re-entry 1 cm proximal to the previous access site. In real-world practice, Applegate et al. reported safe, early re-stick after initial AngioSeal closure in 181 patients within 90 days post-deployment; 80 patients underwent re-stick within 7 days after AngioSeal placement [30].

Univariate analysis identified pre-procedural thrombocytopenia (platelet count ≤ 80 × 10^9^/L) and coagulopathy (INR > 1.5) as significant risk factors for access-site complications (both *p* = 0.034). On multivariate analysis, pre-procedural thrombocytopenia (platelets ≤ 80 × 10^9^/L) was identified as the strongest independent predictor of complications (OR 7.28, 95% CI 1.51–35.12; *p* = 0.013). While VCDs like AngioSeal provide mechanical closure (sandwiching the arteriotomy), the initial formation of a stable hemostatic plug relies heavily on platelet aggregation. Pre-procedural INR > 1.5 showed a trend towards increased risk though it did not reach statistical significance in this model (OR 2.78, 95% CI: 0.86–9.00; *p* = 0.089). Kim et al. showed that smoking (OR 3.50, 95% CI 2.00–6.05, *p* ≤ 0.001), use of antiplatelet therapy (OR 2.01, 95% CI 1.04–3.87, *p* = 0.037) and use of heparin (OR 1.78, 95% CI 1.10–2.86, *p* = 0.018) were independently associated with higher complication rates [7]. While our overall results support immediate sheath removal after emergent embolization, our study identified specific high-risk cohorts that warrant careful management. Our data suggests that for patients with significant thrombocytopenia, the mechanical security of a VCD may be insufficient, and these patients may benefit from prolonged manual compression or delayed sheath removal once platelet counts have been corrected. Similarly, patients with pre-procedural INR > 1.5 showed a trend toward increased risk for both reintervention and access site complications. While statistical significance in the multivariate model was not met, a strategy of delayed sheath removal remains a viable option in this group of patients, who may be at risk of both reintervention and access site complications.

This study has several limitations. Its retrospective, single-center design limits generalizability to institutions with different patient populations, operator expertise, ultrasound-guided access protocols, or post-procedure care pathways. Because immediate sheath removal after emergency embolization was institutional standard practice throughout the study period, no contemporaneous control group with delayed sheath removal was available for direct comparison—an important limitation when assessing the relative safety of the two strategies.

As discussed, VCD selection was at the operator’s discretion and introduced confounding by indication bias. Procedurists likely favored fully extravascular devices (e.g., Mynx Control) or MC in cases perceived to be at higher risk of re-bleeding. While we did find that the choice of VCD used was a significant independent predictor of reintervention and access site complication on multivariate analysis, a conclusion cannot be made.

Patients who died within 7 days from non-access-site causes (*n* = 7) were excluded to allow meaningful assessment of reintervention and delayed complications. However, this introduces a survival bias by potentially excluding the most critically ill patients who may have had the most profound coagulopathy and highest risk of access site failure.

As a retrospective analysis, the study relied on the accuracy and consistency of electronic medical records, and minor complications documented by multiple clinicians and nursing staff may have been under-reported or inconsistently classified. Additionally, despite being a relatively large series, the absolute number of reinterventions and access site complications remain low. Consequently, our multivariate analysis may be underpowered to detect smaller effect sizes for certain variables (e.g., INR), representing a potential Type II error. Finally, patient-reported outcomes (comfort, time to ambulation) and formal cost-effectiveness analyses were not performed. Prospective, randomized multicenter studies are needed to definitively compare immediate versus delayed sheath removal strategies and to better define risk stratification in coagulopathic or antithrombotic-treated patients.

## 5. Conclusions

Immediate femoral sheath removal with ultrasound guidance following emergency embolization is a safe and effective practice with low rates of re-intervention and complications in the majority of patients. Immediate sheath removal in this patient population could lead to improved patient comfort, early ambulation, reduced hospital costs. That said, patients with preprocedural coagulopathy, particularly pre-procedural thrombocytopenia (platelets ≤ 80 × 10^9^/L), are at significant risk of access site complications. We recommend a risk-stratified approach, and high-risk patients may benefit from delayed sheath removal.

## Figures and Tables

**Table 1:** Reintervention rates by patient and procedural factors.

Factors	Total (*n* = 322)	Reintervention		*p*-Value
		Yes (*n* = 13)	No (*n* = 309)	
Indication				0.915
Upper GI hemorrhage (%)	62 (19.3)	4 (6.5)	58 (93.5)	
Lower GI hemorrhage (%)	85 (26.4)	2 (2.4)	83 (97.6)	
Hepatic hemorrhage (%)	28 (8.7)	1 (3.6)	27 (96.4)	
Splenic hemorrhage (%)	39 (12.1)	2 (5.1)	37 (94.9)	
Renal hemorrhage (%)	30 (9.3)	1 (3.3)	29 (96.7)	
Hemoptysis (%)	28 (8.7)	2 (7.1)	26 (92.9)	
Pelvic hemorrhage (%)	25 (7.8)	0 (0.0)	25 (100.0)	
Lumbar artery bleeding (%)	9 (2.8)	1 (11.1)	8 (88.9)	
Inferior epigastric artery (%)	7 (2.2)	0 (0.0)	7 (100.0)	
Intercostal artery (%)	4 (1.2)	0 (0.0)	4 (100.0)	
Extremity hemorrhage (%)	3 (0.9)	0 (0.0)	3 (100.0)	
Polytrauma (%)	2 (0.6)	0 (0.0)	2 (100.0)	
Embolisation				0.323
Performed (%)	296 (91.9)	11 (3.7)	285 (96.3)	
Not performed (%)	26 (8.1)	2 (7.7)	24 (92.3)	
Closure				0.238
AngioSeal (%)	232 (72.0)	6 (2.6)	226 (97.4)	
Mynx Control (%)	60 (18.6)	5 (8.3)	55 (91.7)	
Perclose Proglide (%)	5 (1.6)	0 (0.0)	5 (100.0)	
Starclose SE (%)	1 (0.3)	0 (0.0)	1 (100.0)	
Manual compression (%)	24 (7.5)	2 (8.3)	22 (91.7)	
Antithrombotic drug use				0.061
Present (%)	119 (37.0)	8 (6.7)	111 (93.3)	
Absent (%)	203 (63.0)	5 (2.5)	198 (97.5)	
Preprocedural Hb ≤ 100 g/L				0.286
Present (%)	202 (62.7)	10 (5.0)	192 (95.0)	
Absent (%)	119 (37.0)	3 (2.5)	116 (97.5)	
Preprocedural PLT ≤ 80 × 10^9^/L				0.647
Present (%)	16 (5.0)	1 (6.3)	15 (93.8)	
Absent (%)	305 (94.7)	12 (3.9)	293 (96.1)	
Preprocedural INR > 1.5				0.105
Present (%)	33 (10.2)	3 (9.1)	30 (90.9)	
Absent (%)	273 (84.8)	9 (3.3)	264 (96.7)	
Preprocedural GFR < 60 mL/min				0.647
Present (%)	118 (36.6)	4 (3.4)	114 (96.6)	
Absent (%)	203 (63.0)	9 (4.4)	194 (95.6)	

GI, gastrointestinal; Hb, hemoglobin; PLT, platelet; INR, international normalized ratio; GFR, glomerular filtration rate.

**Table 2:** Overall complication rates by patient and procedural factors.

Factors	Total (*n* = 322)	Overall Complications		*p*-Value
		Yes (*n* = 20)	No (*n* = 302)	
Sheath size				0.789
4 French (%)	10 (3.1)	0 (0.0)	10 (100.0)	
5 French (%)	293 (91.0)	19 (6.5)	274 (93.5)	
6 French (%)	14 (4.3)	1 (7.1)	13 (92.9)	
7 French (%)	5 (1.6)	0 (0.0)	5 (100.0)	
Closure				0.106
AngioSeal (%)	232 (72.0)	12 (5.2)	220 (94.8)	
Mynx Control (%)	60 (18.6)	8 (13.3)	52 (86.7)	
Perclose Proglide (%)	5 (1.6)	0 (0.0)	5 (100.0)	
Starclose SE (%)	1 (0.3)	0 (0.0)	1 (100.0)	
Manual compression (%)	24 (7.5)	0 (0.0)	24 (100.0)	
Antithrombotic drug use				0.442
Present (%)	119 (37.0)	9 (7.6)	110 (92.4)	
Absent (%)	203 (63.0)	11 (5.4)	192 (94.6)	
Preprocedural Hb ≤ 100 g/L				0.779
Present (%)	202 (62.7)	12 (5.9)	190 (94.1)	
Absent (%)	119 (37.0)	8 (6.7)	111 (93.3)	
Preprocedural PLT ≤ 80 × 10^9^/L				0.034 *
Present (%)	16 (5.0)	3 (18.8)	13 (81.3)	
Absent (%)	305 (94.7)	17 (5.6)	288 (94.4)	
Preprocedural INR > 1.5				0.034 *
Present (%)	33 (10.2)	5 (15.2)	28 (84.8)	
Absent (%)	273 (84.8)	15 (5.5)	258 (94.5)	
Preprocedural GFR < 60 mL/min				0.26
Present (%)	118 (36.6)	5 (4.2)	113 (95.8)	
Absent (%)	203 (63.0)	15 (7.4)	188 (92.6)	

Hb, hemoglobin; PLT, platelet; INR, international normalized ratio; GFR, glomerular filtration rate. * *p* value of <0.05 is considered to be significant.

## Data Availability

The data supporting the findings of this study are available upon reasonable request from the corresponding author T.H. via email. Due to confidentiality agreements and institutional policies protecting sensitive patient information from the hospital where the study was conducted, the data cannot be made publicly available. Requests will be evaluated to ensure compliance with ethical and legal standards.

## Abbreviations

The following abbreviations are used in this manuscript:

BMI	Body Mass Index
CFA	Common Femoral Artery
CI	Confidence Interval
CIRSE	Cardiovascular and Interventional Radiological Society of Europe
CT	Computed Tomography
EUS-FNA	Endoscopic Ultrasound-guided Fine Needle Aspiration
GDA	Gastroduodenal Artery
GFR	Glomerular Filtration Rate
GI	Gastrointestinal
Hb	Hemoglobin
ICU	Intensive Care Unit
INR	International Normalized Ratio
IQR	Interquartile Range
MC	Manual Compression
OR	Odds Ratio
PLT	Platelet Count
PSPDA	Posterior Superior Pancreaticoduodenal Artery
US	Ultrasound
VCD	Vascular Closure Device
